# Molybdenum–Carbon Xerogel Composites for ORR-Based Electro-Catalytic Applications

**DOI:** 10.3390/gels12070617

**Published:** 2026-07-09

**Authors:** Luis A. Cavazos-Cuello, Abdelhakim Elmouwahidi, Esther Bailón-García, Jacob Josafat Salazar Rábago, Francisco Carrasco-Marín, Agustín F. Pérez-Cadenas

**Affiliations:** 1 Materiales Polifuncionales Basados en Carbono (UGR-Carbon), Dpto. Química Inorgánica, Unidad de Excelencia Química Aplicada a Biomedicina y Medioambiente, Universidad de Granada, ES18071 Granada, Spain; luiscacl97@correo.ugr.es (L.A.C.-C.); estherbg@ugr.es (E.B.-G.); fmarin@ugr.es (F.C.-M.); 2 Facultad de Ciencias Químicas, Universidad Autónoma de Nuevo León, Av. Universidad S/N, Cd. Universitaria, San Nicolás de los Garza 66455, N.L., Mexico; jacob.salazarrb@uanl.edu.mx

**Keywords:** carbon xerogel, Mo_2_C, ORR, electro-Fenton, molybdenum

## Abstract

Molybdenum-doped xerogel composites were prepared and applied in the electro-degradation of tetracycline (TTC), an antibiotic commonly prescribed for the treatment of bacterial infections. Xerogels containing 1, 6, and 14 wt% Mo were synthesized using an RF sol–gel polymerization method in cylindrical molds and were subsequently characterized in terms of their textural, chemical, and electrochemical properties, focusing on the oxygen reduction reaction (ORR). Textural characterization revealed well-developed surface areas and mesoporosity. Electrochemical analysis showed that Mo loading plays a vital role in the ORR mechanism: lower metal content favors the four-electron pathway with lower hydrogen peroxide selectivity, whereas higher Mo loadings promote bifunctional behavior, enabling both in situ H_2_O_2_ generation and hydroxyl radical production. Undoped and doped xerogels were very active for TTC degradation via the electro-Fenton process, but the presence of MoO_3_ and Mo_2_C phases improved up to 12% in removal efficiency after 480 min of treatment.

## 1. Introduction

Water contamination through emerging pollutants is a worldwide problem that demands the research and design of novel materials to achieve more efficient treatments, as well as reduce operating costs. One class of pollutants that has gained significant attention during the last decades is pharmaceutical compounds. Since human bodies do not metabolize the entire ingested dose, these compounds are excreted through the kidneys into urine and then into municipal sewage systems, eventually being discharged to surface water bodies in their active form, where they interact with unwanted aquatic species and disrupt environmental processes at concentrations as low as pmol/L [1].

Among the pharmaceutical compounds discharged to wastewaters, antibiotics pose the biggest threat to the environment, since their constant contact with surface water bodies can lead to increasingly resistant bacteria that can potentially proliferate in the environment, facilitating infections that are harder to treat, making them a menace not only to environmental life, but to human life as well [2]. Regarding antibiotics, tetracycline hydrochloride (TTC) stands out since it is one of the most frequently prescribed antibiotics used to treat stomach and respiratory infections and has been detected from a wide range of sources, such as hospital effluents and sewage systems, among many others, with treatment plants detecting influent concentrations as high as 1300 ng/L, making its removal from wastewater a priority for the future of our society [3,4,5].

Many technologies have been employed to remove TTC from wastewater, such as membrane filtration and chemical precipitation processes. However, these are associated with high operational and maintenance costs related to pumping demands and constant membrane regeneration, as well as slurry formation from precipitation processes. Advanced Oxidation Processes (AOPs) stand out due to their ease of implementation, as well as their proven efficiency in eliminating antibiotics like TTC from wastewater [6,7].

AOPs are chemical processes based on the generation of highly reactive species, commonly referred to as reactive oxygen species (ROS), which will in turn degrade the target molecule to simpler, less toxic structures. One of the most commonly generated species is the hydroxyl radical (⋅OH), which is produced through diverse methodologies such as photocatalysis and sonocatalysis, both of which use electromagnetic radiation in the form of light and sound for the generation of radicals. These, however, have energetic limitations when implemented in large-scale operations, limiting their applicability [8].

One of the most common ROS generation methods is the Fenton process, where iron cations are cyclically oxidized and reduced between their +2 and +3 oxidation states in the presence of H_2_O_2_ for the generation of (⋅OH) species. The traditional Fenton process has the advantage of highly efficient generation of radicals, as well as its ease of implementation; however, its greatest limitation is the industrial handling of hydrogen peroxide, which implies operational hazards, as well as a very limited pH range of operation, usually around three, while also favoring iron slurry formation, which requires difficult posterior treatments [9].

To combat these limitations, electrically assisted AOPs (often referred to as eAOPs), such as the electro-Fenton process, are introduced. These have the advantage of in situ generation of H_2_O_2_ at the cathode through the reduction of oxygen present in the medium, and can be applied with a wide variety of metals, such as iron (electro-Fenton) [10] as well as transition metals (electro-Fenton-like) [11].

As previously mentioned, the rate-limiting step in the electro-Fenton process is the reduction of oxygen at the cathode surface to form hydrogen peroxide (referred to as the ORR, or oxygen reduction reaction). This process can occur via different electron transfer routes, with the two-, three-, and four-electron transfer routes being presented in the following equations [12].

(1) $$O_{2}+2H^{+}+2e^{-}\to H_{2}O_{2}$$

(2) $$O_{2}+2H^{+}+3e^{-}\to \;\cdot OH+OH^{-}$$

(3) $$O_{2}+4H^{+}+4e^{-}\to \;2H_{2}O$$

Among these routes, the two- and three-electron transfer routes are favorable for the degradation of pollutants in wastewater systems, providing hydrogen peroxide, which will transform into radical species, as well as the direct production of said radicals through the three-electron route, while the four-electron transfer route produces mostly water, favoring energy storage applications such as supercapacitors [13]. The challenge lies within favoring the two-electron transfer route while simultaneously minimizing the four-electron transfer route; hence, special attention must be provided to the design and implementation of the cathode catalyst to maximize 2e^−^ ORR activity, as well as having a pore structure such that pollutant molecules can easily diffuse and adsorb on the catalyst surface to favor electro-degradation.

Within the realm of ORR catalysts, carbon-based materials stand out due to their high mechanical stability, as well as their developed surface area and good electric conductivity. However, despite these properties, chemical kinetic processes tend to be slow when compared to traditional cathodes such as Pt or Pd, which have higher electrical conductivities than carbon-based materials [14].

To address this issue, transition metals are incorporated into the active sites of the catalyst to increase electrochemical activity. There are two methodologies for this: the first consists of synthesizing a blank material and chemisorbing metallic particles onto the surface of the material through the incipient wetness impregnation method [15], while the second involves the inclusion of a metallic precursor during the synthesis step of the material, which reduces the limitations associated with the incipient wetness impregnation method, such as cluster formation and lixiviation of the metal, and increases its dispersion not only on the surface of the material but throughout its entire bulk.

In recent decades, one of the most versatile carbon-based materials developed has been carbon xerogels, which are synthetic carbon materials prepared through the polycondensation of organic monomers, traditionally resorcinol (R) and formaldehyde (F). The resulting material possesses elevated surface areas and developed porosity, with the advantage of an easily modifiable surface and electric properties through the addition of chemical compounds such as heteroatoms or transition metals to maximize their efficiency in any needed application [16,17,18]. Thus, the use of these types of materials has shown interesting applications for TTC elimination via the electro-Fenton process in the aqueous phase, eliminating the need to add the Fenton reagent to the reaction medium for ROS generation [19].

In the present work, we develop carbon xerogel–molybdenum composites with the aim of their application in the above-mentioned reactions. There are very few studies in the literature on the development of Mo-doped carbon xerogels, and none with the high metal loading incorporated here; moreover, the few existing studies do not use them in wastewater treatment applications.

The first study regarding carbon gels doped with Mo was published in 2005 [20], showing molybdenum carbide formation after high-temperature reduction treatments, which is a very interesting Mo phase for ORR applications. In this work, Mo was incorporated during the R-F polymerization process. Later, other preparation methods of similar materials were published, but they always refer to very low Mo loadings [21,22].

Recently, a new advance in the application of carbon-based gels for the degradation of TTC using the electro-Fenton process was the direct production of hydroxyl radicals via a three-electron transfer route [23]. In the same application, carbon gel microspheres coated with manganese showed bifunctional characteristics, producing hydroxyl radicals through in situ generation of H_2_O_2_, achieving 90% degradation after 300 min of treatment [24]. Iron carbide–carbon gel composites have also shown excellent electrochemical behavior in this same TTC electro-degradation reaction [10].

As seen in the background research, there are very few works in the literature where Mo_2_C/carbon phases are used for ORR applications, with the Mo phase always being supported on the carbon matrix rather than forming part of a composite material [22,25]. With this in mind, the objective of this study is to develop carbon xerogel–molybdenum composites with high Mo loading, suitable for electro-catalytic applications. The focus is on the ORR possibilities depending on the reaction mechanism, that is, (i) energy storage or (ii) electro-Fenton degradation for pollutants like TTC from wastewater.

## 2. Results and Discussion

### 2.1. Morphological, Textural and Surface Chemistry Characterization

**Figure 1:**
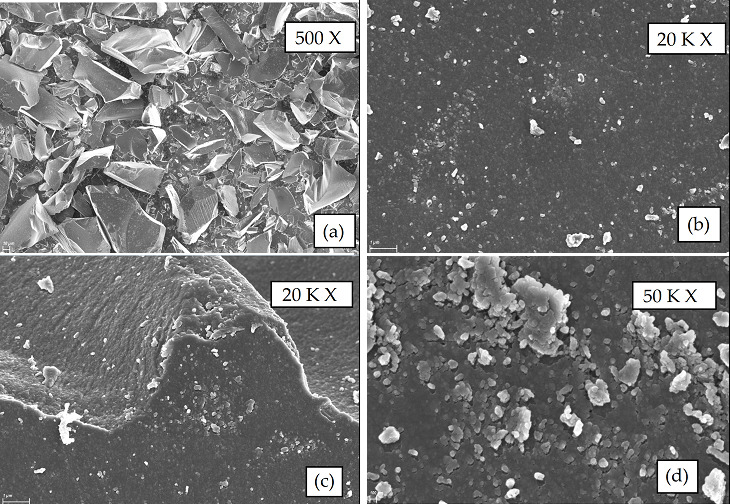
SEM micrographs for XMo1 at different magnifications: (**a**) 500 X, (**b**,**c**) 20 K X, (**d**) 50 K X.

The morphological characterization of the 1% Mo xerogel by SEM is presented in Figure 1a–d at different magnifications. In these images, a very rough surface typical of carbon xerogels can be observed. However, a significant difference is that Mo-containing xerogels reported in the literature are typically found in the form of microspheres, whereas the surface observed in these xerogels appears much more compact with small indentations. These features are ideal for applications involving the formation of monolithic parts for contaminant removal by degradation processes, being more resistant from a mechanical perspective [26].

**Figure 2:**
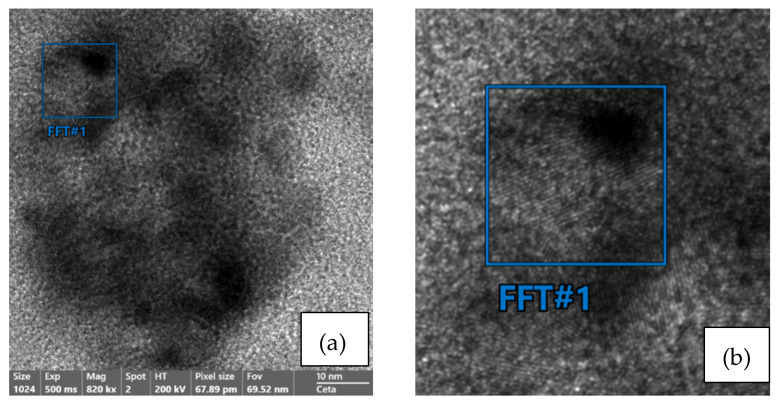
HRTEM micrographs for XMo6. The (**a**) corresponds to the HRTEM micrograph of XMo6, while (**b**) shows the enlarged region highlighted in blue in (**a**).

The surface morphology of the Mo-doped xerogels was studied using HRTEM for the XMo6 xerogel, with the micrographs being presented in Figure 2a,b. In these, a striated pattern corresponding to the formation of crystal structures was observed; this pattern was identified throughout the entire surface of the material. Presumably, the crystal structures formed are associated with the formation of Mo_2_C and MoO_3,_ which are the most reported phases for carbon structures containing Mo [20].

**Figure 3:**
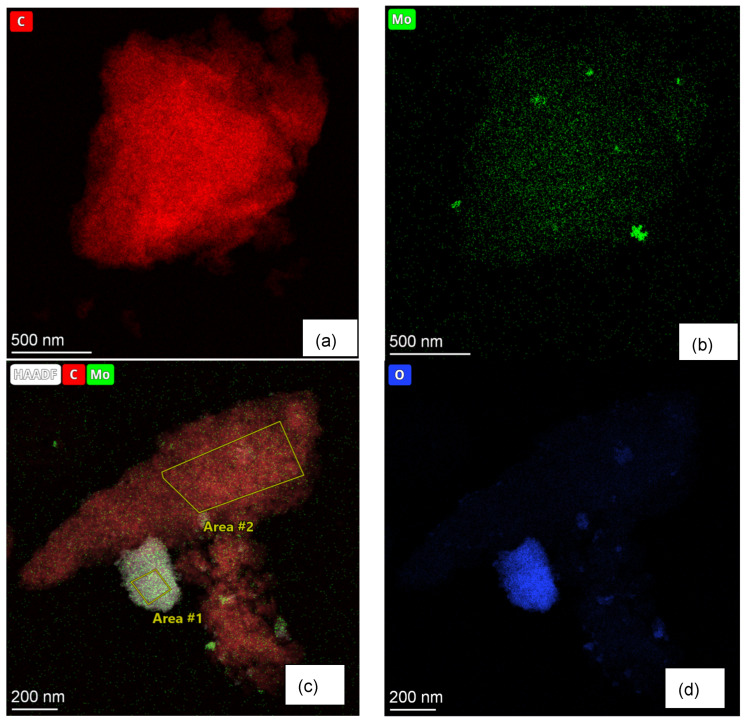
HAADF image and corresponding elemental maps of the XMo6 surface, showing the distribution of C, O, and Mo. (**a**) HAADF image of the XMo6 sample and the corresponding elemental map showing the distribution of C. (**b**) HAADF image of the XMo6 sample and the corresponding elemental map showing the distribution of O. (**c**) HAADF of the XMo6 sample with the corresponding overlaid elemental maps of C (area #2) and Mo (area #1). (**d**) HAADF image of the XMo6 sample and the corresponding elemental map showing the distribution of Mo.

Additionally, HAADF scanning images were obtained for the same XMo6 sample to observe the molybdenum distribution on the surface of the material. These images are presented in Figure 3a–d for the C, O and Mo micrographs. Due to the starting materials in the xerogel synthesis being carbon-based monomers, it is expected to find a wide coverage of carbon throughout the entire surface like it is observed in Figure 3a,c.

The HAADF image in Figure 3b clearly shows a very uniform distribution of molybdenum throughout the carbonaceous matrix. Some small agglomerations of Mo particles are also detected, which are shown at higher resolution in Figure 3c; these are commonly observed in this type of metal–carbon composite material when the metal content is significant, as is the case here. On the other hand, the oxygen distribution shown in Figure 3d does not fully coincide with the Mo-rich regions. This observation may indicate the presence of a molybdenum carbide (Mo_2_C) phase.

**Figure 4:**
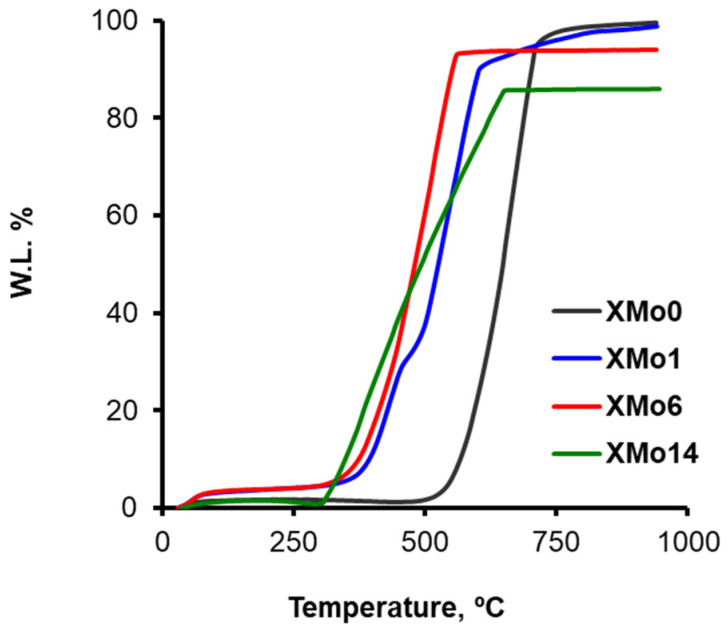
TGA profiles for the prepared xerogels.

The TGA curves for the molybdenum-containing xerogels are presented in Figure 4. Based off the weight loss, the real metal content was calculated by assuming the residual mass corresponds to molybdenum oxide. The actual weight percentages obtained for XMo1, XMo6 and XMo14 are 1.23, 5.99 and 14.10%, respectively. Given that the Mo percentages were close to the theoretical value, both the synthesis procedure and incorporation method can be considered successful.

**Figure 5:**
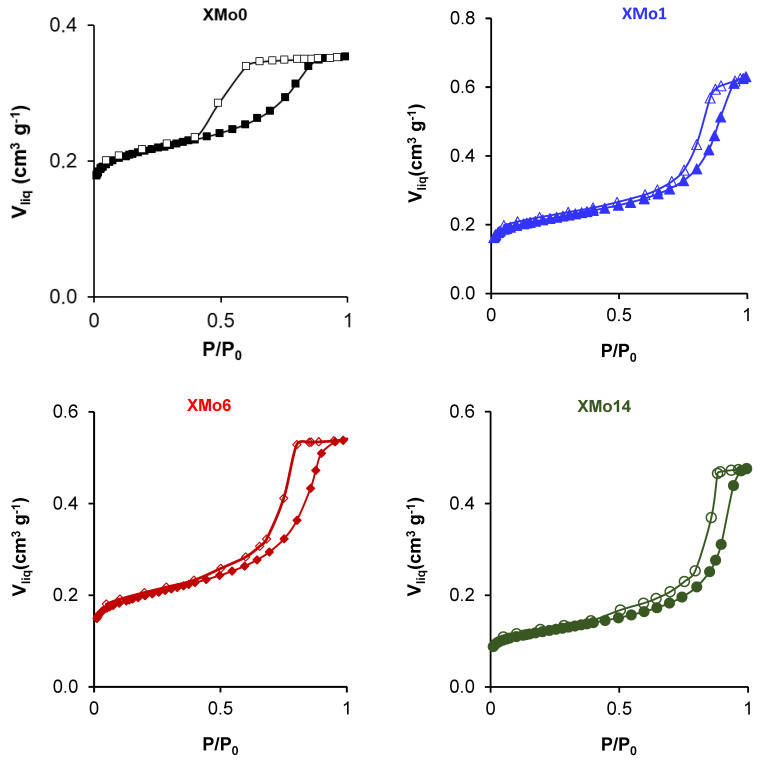
N_2_ isotherms for the prepared materials.

The textural properties of the obtained xerogels were analyzed by nitrogen adsorption–desorption isotherms, which are presented in Figure 5. The isotherms for all the xerogels are classified as hybrid type I and IV according to the International Union of Pure and Applied Chemistry classification [27], which is characteristic of materials exhibiting a combination of microporosity and mesoporosity, as is the case with many carbon materials like carbon xerogels.

All the isotherms show a sharp increase at low relative pressures (P/P_0_ < 0.05), associated with the rapid filling of the micropores. In addition, all the isotherms display H2-type hysteresis, characteristic of typical materials with variable pore size and non-defined morphologies, which is the case for the prepared molybdenum xerogels.

**Figure 6:**
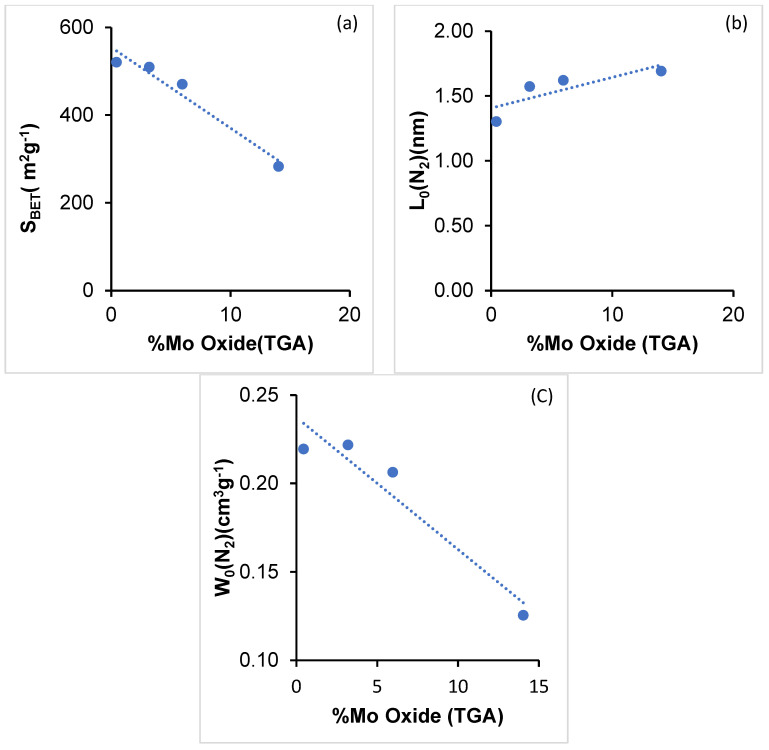
Effect of Mo loading on the textural properties (**a**) surface area (S_BET_); (**b**) mean pore width L_0_(N_2_) and (**c**); micropore volume W_0_(N_2_) of the prepared xerogels.

The textural properties of the xerogels are summarized in Table 1, which shows the BET area, the micro- and mesopore volumes and the average pore width, where a specific area of 520 m^2^/g can be observed for the XMo0 material, evidencing a well-developed surface area commonly found in carbon xerogels. However, a general decrease in surface area can be observed when the metal content increases. The microporosity was not heavily affected until reaching very high Mo content (XMo14) and the strong effect that the Mo had on the mesoporosity development should be remarked, since all Mo-doped samples show much higher mesopore volumes than XMo0 xerogel. This fact is very important from the perspective of the application of these materials in catalytic reactions in the liquid phase. The presence of Mo clearly influences the RF polymerization process, resulting in smaller primary particles, which, after merging, form pores in the mesopore range. Figure 6 shows the variations in different textural parameters with the variations in the Mo content, supporting the above-mentioned effect. Microporosity also is clearly affected by the Mo loading in the RF mixtures developed to form organic gels.

Texturally speaking, all the xerogels obtained in this study are suitable for pollutant removal through tertiary processes and electro-degradation, as they enable easy diffusion of reactants to active sites, as well as facilitating the charge transport required for the reduction of the oxygen in the medium to produce ROS.

**Figure 7:**
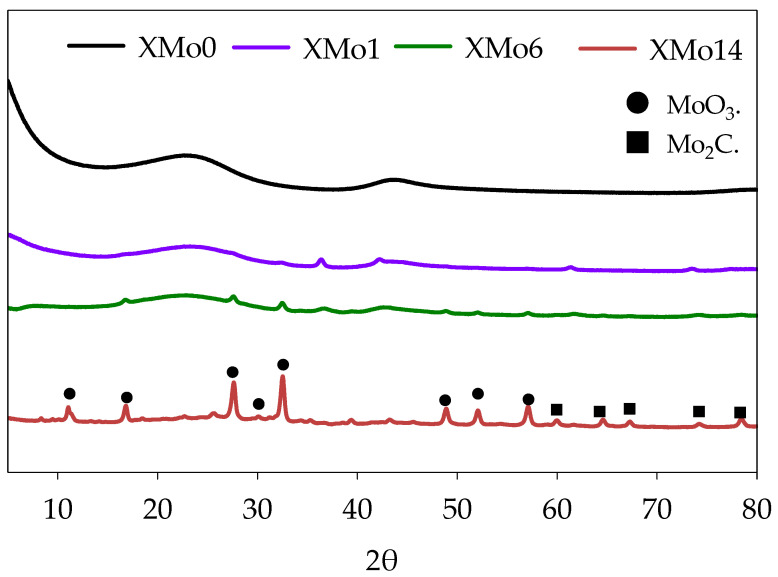
X-ray diffraction spectra for the Mo-loaded xerogels.

The X-ray diffraction patterns for the molybdenum xerogels can be observed in Figure 7, where no crystal structures were identified for the blank material, which is expected behavior. However, the graphite reflexes were identified for all the materials in the range of 25–45 2θ, with XMo14 having these reflexes attenuated, possibly due to the molybdenum forming chemical bonds with the carbon matrix of the xerogel. No crystal structures were identified for the materials XMo1 and XMo6; this could be due to the formation of amorphous molybdenum-based phases or small, flat particles with very little thickness and therefore low crystallinity.

However, the XMo14 xerogel presented a clear diffraction pattern associated with crystalline Mo structures, specifically with MoO_3_ and Mo_2_C in their orthorhombic forms (Crystallography Open Database entries 1011043 and 1539795, respectively), signaled as circles for MoO_3_ and squares for Mo_2_C in Figure 7.

**Figure 8:**
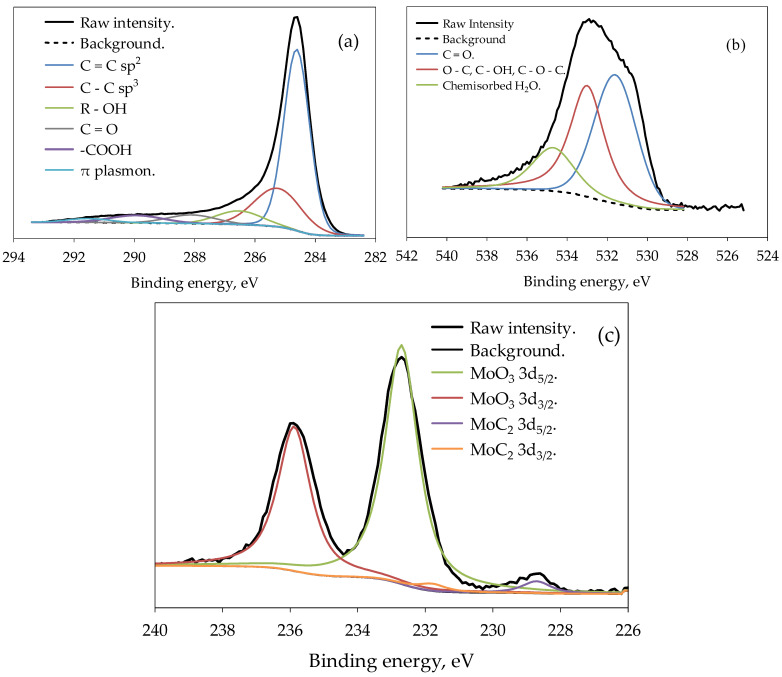
Deconvoluted XPS spectra for the XMo6 material for (**a**) C1s, (**b**) O1s and (**c**) Mo3d.

The XPS analysis was performed by deconvolving the peaks obtained through analyzing the C1s, O1s and Mo3d regions. Figure 8 shows the deconvoluted XPS spectra for the XMo6 material for (a) C1s, (b) O1s and (c) Mo3d, with the deconvoluted spectra for the rest of the materials being presented in Figures S1–S3 (Supplementary Data), whilst Table S1 (Supplementary Data) presents the main parameters obtained through the deconvolution of the peaks obtained.

For the C1s region, several bands were identified, with binding energies around 284.6, 285.2, 286.5, 288.6, 290.0 and 291.5 eV corresponding to six functional groups, C=C sp^2^, C–C sp^3^, R–OH, C=O, COOH and π plasmon, which are commonly found in carbon structures [28,29,30,31]. For the O1s region, binding energies around 531, 533 and 534 eV, corresponded to the C=O, O–C, C–OH, and C–O–C groups, as well as chemisorbed water from the environment in which the samples were analyzed.

The Mo3d region shows two main Mo species found at the 3d_5/2_ binding energies of 228.60 and 232.81 eV. The first one corresponds to Mo_2_C 3d_5/2_ with its corresponding 3d_3/2_ peak in 231.8 eV; after integration, this phase contemplates around 6% of all the molybdenum found in the surface of the XMo6 sample. Meanwhile, the second peak corresponds to MoO_3_ 3d_5/2_, with its corresponding 3d_3/2_ peak in 235.98 eV, making up most of the Mo present in the surface of all the doped xerogels.

Both Mo phases obtained through the XPS deconvolution are in accordance with what was found in the XRD analysis, with the peak designation being based on literature research [21,32]. Nevertheless, the Mo_2_C content identified in the external surface of the XMo14 sample was negligible.

Additionally, the surface analysis of the samples carried out by XPS is collected in Table 2. A decrease in the carbon content after molybdenum insertion in the samples is observed. Nevertheless, the superficial Mo contents in the XMo6 and XMo14 samples are much smaller than those obtained in the TGA analysis, indicating the dispersion of the metal throughout the xerogel matrix, as most of this molybdenum is probably inaccessible for catalytic applications.

### 2.2. Electrochemical Characterization

The electrochemical characterization of the synthesized carbon xerogels consisted of cyclic and linear sweep voltammetry analyses under oxygen- and nitrogen-saturated conditions, using a 0.1 M KOH electrolyte for the oxygen reduction reaction (ORR).

**Figure 9:**
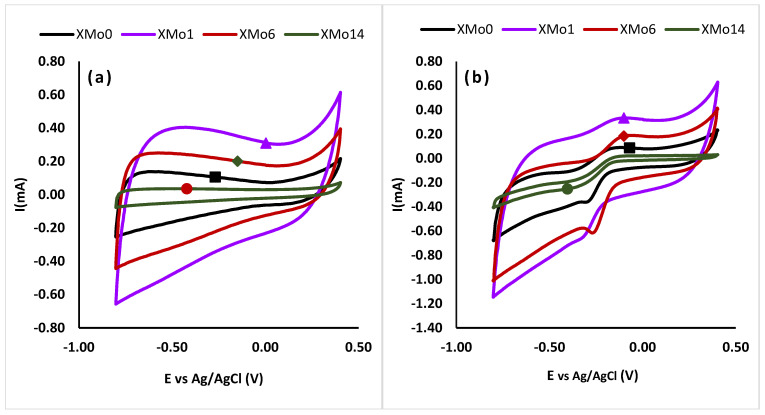
Cyclic voltammetries for the synthetized xerogels in (**a**) N_2_ and (**b**) O_2_.

The cyclic voltammograms of the prepared xerogels are presented in Figure 9, where 9a corresponds to the experiments carried out under inert N_2_-saturated conditions, while 9b represents the experiments under O_2_ saturation. For the nitrogen-saturated experiments, capacitive behavior is observed with no undesired reactions present. Additionally, the voltammograms show a small variation in the generated current between the anodic and cathodic regions.

In the case of oxygen-saturated experiments, the presence of the ORR is clearly observed due to the faradaic response obtained, which is especially emphasized for the sample without Mo content. All the samples prepared show an increase in electrochemical activity at a potential around −0.2 V, indicative of the oxygen reduction reaction taking place.

For both N_2_ and O_2_ experiments, an increase in generated current is observed when increasing Mo loading from 0 to 1%, associated with the active phases found in the XRD and XPS characterizations, followed by a decrease in generated current as the metal content increases. This behavior is attributed to the decrease in surface area, as well as micro and mesopore volumes found through the N_2_ adsorption isotherms.

**Figure 10:**
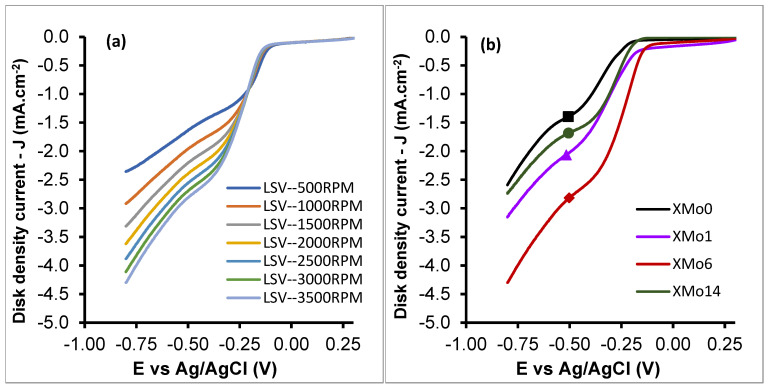
LSV at different rotation speeds for XMo6 (**a**) and comparison between materials (**b**).

To further analyze the ORR, the xerogels were evaluated using linear sweep voltammetry (LSV) tests, which consist of applying an electric potential only in the reduction scan to study the generated current under different experimental conditions. Figure 10a shows the LSV analysis for the XMo6 xerogel at different rotation speeds (RPM) of the rotating ring–disk electrode, while Figure 10b presents the LSV curves at 3500 RPM for the Mo-containing xerogels.

The behavior shown in Figure 10a demonstrates an increase in the generated current as the rotation speed (RPM) of the rotating ring–disk electrode increases. This behavior is expected, since increasing the electrode rotation speed reduces mass transfer resistance in the system due to the decrease in the thickness of the boundary layer at the solid–liquid interface. On the other hand, for Figure 10b, an increase in generated current is observed for the doped xerogels. In addition, the presence of Mo_2_C could be related with better electro-catalytic behavior towards an oxygen reduction reaction with high H_2_O_2_ formation, while sample XMo1 shows good electro-chemical characteristics for fuel cell applications, such as a high J_k_ value and transferred electrons. The combined effect of low Mo doping with high accessibility to well-developed mesoporosity (Table 1) could be the key to this phenomenon.

**Figure 11:**
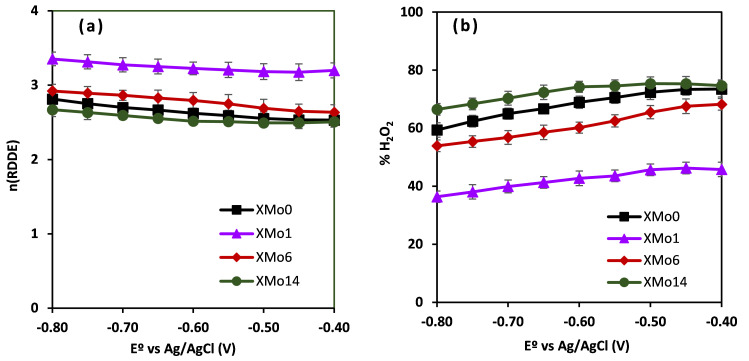
Number of electrons transferred (**a**) and H_2_O_2_ selectivity (**b**) for the Mo-doped xerogels. Experimental points represent the average between three experiments, while error bars are calculated as ($\bar{x}\pm \sigma$).

To further understand the mechanisms involved in the ORR, the trends in the number of electrons transferred and H_2_O_2_ selectivity were obtained using the Koutecky–Levich and RDDE equations for *n* and %H_2_O_2_; the result of this is shown in Table 3 for −0.8 V and in Figure 11 for the potential range of −0.8 to −0.4 V, with the Koutecky–Levich dependance plots being presented in Figure S4 (Supplementary Data).

The above-mentioned different behaviors can be better explained as follows: the XMo1 sample presented the lowest peroxide selectivity (Table 3 and Figure 11), as well as being the closest to the four-electron pathway. This suggests that this material is very suitable for fuel cell applications, and, as such, it will not be evaluated for the electro-Fenton process. On the other hand, the XMo6 and XMo14 samples show a high H_2_O_2_ selectivity, as well as approaching the three-electron transfer route the closest, indicating that they have the capability of not only producing hydrogen peroxide in situ, but also transforming said peroxide directly into ROS, meaning that they are ideal for wastewater treatment applications, such as the electro-Fenton process for emerging pollutants like TTC. This behavior can be explained due to their higher Mo content in general and Mo_2_C phase detected by XPS and XRD.

The results obtained from the electrochemical characterization are consistent with what has been reported in the literature for carbon matrices doped with molybdenum. One example is the study by Huang et al. (2015), in which nanocomposites of graphite and Mo_2_C supported on carbon were synthesized to study the ORR [30]. They reported current densities of 2.3 mA/cm^2^ at −0.6 V, with the number of transferred electrons ranging between 2.1 and 3.2, calculated using the Koutecky–Levich equation.

On the other hand, the study by Mladenović et al. (2020) [22] investigated molybdenum carbide supported on carbon nanotubes and carbon xerogels for the oxygen reduction reaction. They reported Koutecky–Levich current densities of 2.9 and 1.6 mA/cm^2^ for xerogels and nanotubes with Mo_2_C, respectively, along with electron transfer numbers between 3.0 and 2.7 at 0.6 V at 1600 rpm.

Similar results have been found for other carbon-based matrices incorporating molybdenum, with electron transfer numbers typically ranging between two and three [26,33,34], with additional studies being show in Table S2 (Supplementary Data) [35,36], demonstrating the viability of molybdenum-modified carbon materials for the ORR and, consequently, their potential application in the electro-degradation of contaminants in the aqueous phase.

### 2.3. Tetracycline Degradation Tests

For the TTC degradation experiments, the electrodes were first prepared according to the procedure described in the Section 4, and TTC solutions with concentrations higher than 50 mg/L were prepared based on previous adsorption isotherms of each material shown in Figure S5 (Supplementary Data). This was done so that, after reaching adsorption saturation, the concentration in the medium would approach 50 mg/L. Once saturation was achieved, an electrical potential of −0.6 V was applied using 0.5 M sodium sulfate as the electrolyte.

**Figure 12:**
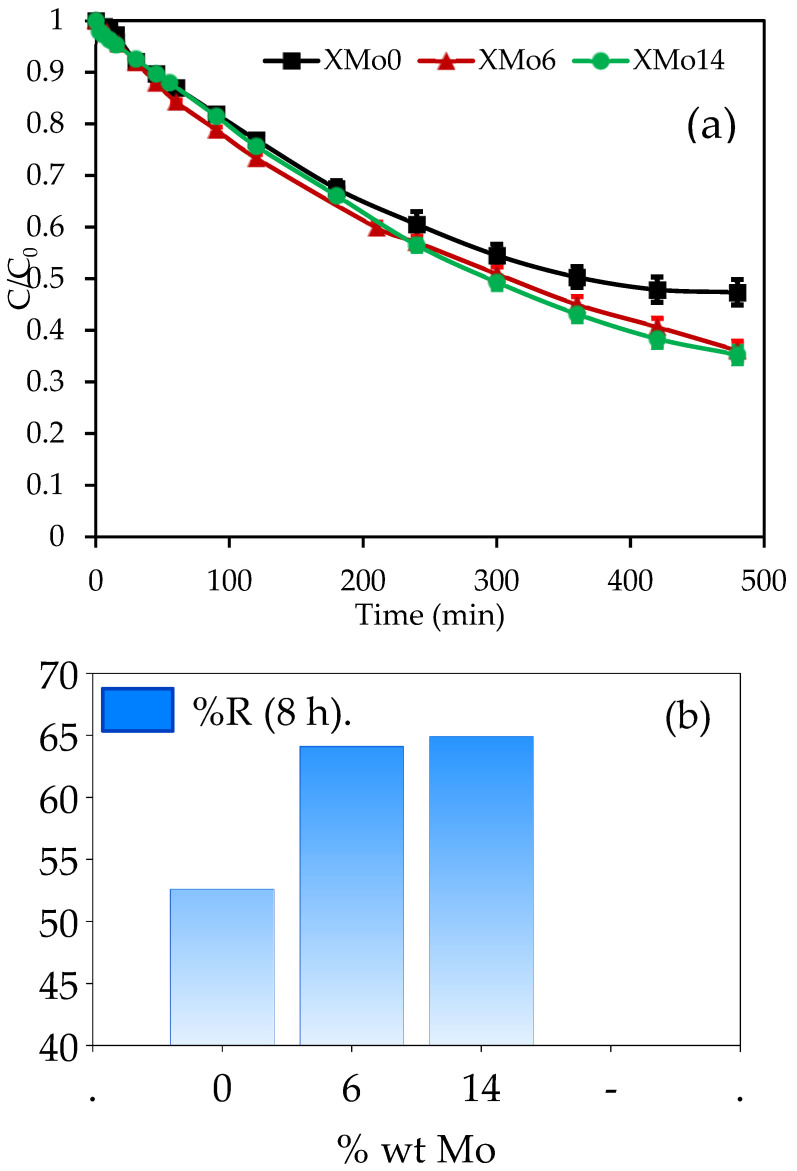
Concentration decay curves for the electro-degradation experiments (**a**), removal percentages obtained (**b**). C_0_ = 50 mg/L post adsorption, pH = 6.5, T = 25 °C. The data points shown are the average between three experiments, with the error bars being calculated as ($\bar{x}\pm \sigma$).

The concentration decay curves are presented in Figure 12a, with the degradation percentages being shown in Figure 12b. It should be remarked that the XMo0 material is able to achieve TTC electro-degradation by reducing oxygen in the medium to radicals. These radicals are adsorbed onto the available active sites and subsequently interact with TTC molecules previously adsorbed, following a Langmuir–Hinshelwood–Hougen–Watson mechanism [33], which is widely reported for carbon-based materials.

As seen in the electrochemical characterization, the XMo6 and XMo14 samples presented the highest current densities, as well as approaching the three-electron transfer route better when compared to the blank material; this is clearly reflected in the higher TTC degradation percentages shown in Figure 12a,b.

The xerogels with higher Mo weight percentages showed higher conversion percentages compared to the other materials, reaching approximately 62% and 64% for the XMo6 and XMo14 xerogels, respectively. This represents an increase of approximately 12% compared to XMo0; this behavior can be attributed to the coexistence of both Mo_2_C and MoO_3_ phases in the Mo-doped xerogels. Although XPS analysis indicates that the Mo_2_C phase is less abundant at the material surface, XRD patterns of all catalysts reveal diffraction peaks corresponding to Mo_2_C, confirming its presence in the bulk structure. According to the literature, Mo_2_C is a highly active phase for the oxygen reduction reaction (ORR) and, consequently, can significantly enhance the electro-Fenton process. Meanwhile, the MoO_3_ phase may contribute additional acidic sites that promote pollutant adsorption and facilitate the generation of reactive oxygen species, further improving catalytic performance.

Additionally, despite the high reduction in apparent surface area of the doped samples when compared to the blank material, it is true that this would also be partially compensated for larger volumes of mesopores present in XMo6 and XMo14. Therefore, the combination of molybdenum phases with the carbon xerogel matrix to form these types of composites materials is very interesting, showing high potential for different electro-chemical applications where the amount of Mo accessibility, as well as the Mo_2_C/MoO_3_ ratio, should be studied in future work for real optimization. The clear effect of mesopore development provoked by the presence of molybdenum during the polymerization process is very interesting since this range of porosity is ideal for liquid-phase reactions in general and, obviously, for electro-Fenton degradation reactions.

## 3. Conclusions

Molybdenum-doped carbon xerogel composites were successfully synthesized using the sol–gel method. The morphological, textural and surface chemical characterization revealed a well-developed pore structure where orthorhombic Mo_2_C and MoO_3_ phases were detected among the samples. The textural characteristics were clearly influenced by the presence of Mo during the RF polymerization process. The electrochemical characterization through ORR revealed that metal content and phases diverge the potential application of the Mo-doped xerogels, with low metal incorporations favoring energy storage applications through water production, and higher molybdenum loadings favoring bifunctional behavior, enabling the in situ production of hydrogen peroxide, as well as radical species, which is ideal for water purification applications. The Mo xerogels demonstrated favorable activity in the electro-Fenton process for TTC, achieving a 62% degradation rate after 480 min of treatment, with an increase of 12% efficiency with respect to the non-doped sample, demonstrating the effect of both the carbon matrix and Mo phases as active sites for ROS production. These findings pose the Mo-doped xerogels as efficient solutions for water bodies contaminated by emerging pollutants like TTC, providing elevated removal efficiencies with relatively low metal contents required for their preparation.

## 4. Materials and Methods

### 4.1. Chemicals Used

The chemical reagents used for the synthesis of the xerogels are resorcinol (99%, Alfa Aesar Ward Hill, Massachusetts, United States), formaldehyde (37%, Sigma-Aldrich, St. Louis, MO, USA) and sodium molybdate (99%, Sigma-Aldrich). On the other hand, the model pollutant used for the electro-Fenton experiments is tetracycline hydrochloride (98% Sigma-Aldrich).

### 4.2. Synthesis of the Carbon Xerogels with Molybdenum

The synthesis procedure of the carbon xerogels is the sol–gel methodology originally proposed by Pekala [34]. For this, a stoichiometric resorcinol-to-formaldehyde (R/F) molar ratio of 0.5 was used, starting from 0.1 mol of resorcinol (R). Sodium molybdate served as the precursor salt and was added in amounts required to achieve theoretical Mo loadings of 1, 6, and 14 wt.% based on preliminary runs where higher Mo contents led to mechanical instability of the materials. A 50% mass loss during xerogel carbonization was assumed for their preparation.

Once obtained, the mixture was poured into cylindrical molds previously sealed at one end with a propane flame. After filling enough volume to form a 13 cm carbon xerogel column, the molds were completely sealed. Gelation was carried out at ambient temperature for 24 h, followed by curing in an oven at 50 °C for 24 h and then at 80 °C for 120 h.

The resulting organic gels were removed from the molds using a Dremel saw and transferred to a larger glass tube for solvent exchange. Acetone exchange was performed twice daily for three days to remove water trapped within the pore structure. The xerogels were then dried at 80 °C for 24 h.

Carbonization was carried out at 900 °C for 30 min under a nitrogen flow of 150 mL/min, using a heating ramp of 5 °C/min. The resulting materials were labeled as XMoY, where Y represents the theoretical Mo content in the xerogel matrix. The undoped material (0 wt.% Mo) was labeled XMo0 and used as a blank reference.

### 4.3. Chemical, Morphological and Textural Characterization

The textural properties of the samples were obtained using nitrogen adsorption–desorption isotherms at 77 K. Prior to analysis, all materials were degassed under high vacuum (10^−6^ mbar) at 120 °C for 12 h to eliminate any residual physisorbed molecules. The surface area was estimated using the Brunauer–Emmett–Teller (BET) method, while the micro- and mesopore volumes were calculated using the BJH and Dubinin–Radushkevich methods, respectively [37,38]. The morphology of the samples was analyzed using an HRTEM microscope Titan FEI G2 with HAADF mode (Thermo Fisher Scientific TALOS F200X, Waltham, MA, USA); for SEM, a LEO (Carl Zeiss, Oberkochen, Germany) GEMINI-1530 microscope was used. The crystalline structure of the samples was analyzed using X-ray diffraction using a Bruker D8 Advance diffractometer (Karlsruhe, Germany) with Cu kα radiation. The surface composition of the samples was studied using the XPS technique with an Escalab 200R system with a Mg kα radiation source using the binding energy of the C1s peak (284.6 eV) as a reference.

### 4.4. Electrochemical Characterization

The procedure for the electrochemical characterization of the samples in this work is based off other similar carbon materials reported in the literature [10,24]. Briefly, the performance of the materials was measured using a Metrohm Autolab PGSTAT101 potentiostat (Metrohm Autolab, Utrecht, The Netherlands) using a rotating disk–ring electrode with a glassy carbon surface as a working electrode. The electrode was prepared starting from a 1/10 Nafion solution, extracting 1 mL of this solution and suspending 5 mg of fine powder of the carbon xerogel, followed by sonicating for 30 min to ensure proper dispersion. A 20 μL aliquot of the final mixture was carefully dropped-cast onto the disk surface of a rotating ring–disk electrode (RRDE) for ORR testing. For the ORR measurements, a standard three-electrode system was employed at an ambient temperature using a Pt sheet as a counter electrode and Ag/AgCl as a reference electrode in 0.1 M KOH as the electrolyte. Cyclic voltammetry (CV) was studied in a potential window from 0.4 to −0.8 V with a potential sweep of 50 mV/s and a rotational velocity of 1000 rpm, pre-saturating the medium with nitrogen or oxygen for 30 min depending on the experiment. Linear sweep voltammetry was studied in a similar fashion to the CV experiments but we adjusted the rotational velocity of the electrode from 500 to 3500 rpm to study the effect of mass transfer resistance. Using the data obtained from LSV, the number of transferred electrons and the peroxide selectivity were calculated using the RDDE electrode equations:

(4) $$n=\frac{4\times I_{D}}{I_{D}+\frac{I_{R}}{N}}$$

(5) $$\%H_{2}O_{2}=100\times \frac{2\times \frac{I_{R}}{N}}{I_{D}-\frac{I_{R}}{N}}$$

where $I_{D}$ and $I_{R}$ represent the generated currents in the disk and the ring, respectively, while N represents the electron collection efficiency of the electrode, 0.249.

The information obtained through the LSV experiments was also fitted to the Koutecky–Levich equation; this model describes the current density generated as parallel resistances between the electron transfer phenomena and mass transfer phenomena:

(6) $$\frac{1}{J}=\frac{1}{J_{K}}+\frac{1}{B\omega^{1/2}}$$

where ω is the electrode’s rotational speed in min^−1^ and B is calculated as follows:

(7) $$B=0.62\times n\times F\times A\times D^{2/3}\times v^{-1/6}\times S$$

In this equation, F is the Faraday constant, 96,485 C/mol, n is the number of electrons transferred per mole of oxygen reduced, D is the oxygen’s diffusivity in water at ambient temperature (1.9 × 10^−5^ cm^2^/s), v is the kinematic viscosity (0.01 cm^2^/s) and S represents oxygen solubility in water (1.2 × 10^−6^ mol/cm^3^).

### 4.5. Electro-Fenton Tests

For the TTC electro-Fenton tests, the procedure began with the preparation of the carbon electrode. The previously obtained material was first pulverized to a fine particle size. A 9:1 weight ratio mixture of carbon material to 60% PTFE was then prepared and mixed thoroughly until a homogeneous paste was obtained. This paste was subsequently dried under infrared radiation for one hour.

After drying, the paste was spread onto 1 × 3 cm graphite sheets, covering an area of 1 × 1 cm. A mass of 25 mg of paste was applied on each side, resulting in a total of 50 mg per electrode.

Prior to the electro-Fenton experiments, the electrode was placed in contact with a TTC solution for 24 h in the dark to ensure adsorption equilibrium between the xerogel and the antibiotic. The solutions were prepared such that the concentration after adsorption was as close as possible to 50 mg/L, using 0.5 M Na_2_SO_4_ as the supporting electrolyte. This value was considered the initial concentration of the experiment.

Once equilibrium was reached, both the solution and the electrode were transferred to a standard three-electrode electrochemical cell connected to a Biologic VMP potentiostat. A platinum wire was used as the counter electrode, and a Ag/AgCl electrode served as the reference.

The solution was then saturated by bubbling oxygen for 30 min. Afterwards, a potential of −0.6 V was applied to initiate the electro-Fenton process. Starting this, samples were periodically taken during an 8 h period, measuring their absorbance through UV–Vis spectrophotometry using previously prepared calibration curves ($R^{2}>0.998$) to interpret the absorbance data. Each degradation experiment was performed three times to check for reproducibility, with the concentration decay data shown being the average of the three experimental runs.

## Figures and Tables

**Table 1:** Textural properties of the xerogels.

Sample	Surface Area (S_BET_), m^2^/g	Micropore Volume (W_0_), cm^3^/g	Average Pore Width (L_0_), nm	Volume Adsorbed (V_0.95_), cm^3^/g	Mesopore Volume (V_meso_), cm^3^/g	%Mo
XMo0	520	0.219	1.300	0.352	0.133	0.00
XMo1	509	0.222	1.570	0.625	0.404	1.23
XMo6	470	0.206	1.620	0.535	0.329	5.99
XMo14	283	0.125	1.692	0.439	0.314	14.10

**Table 2:** Elemental surface composition through XPS analysis.

Sample	%C	%O	%Mo
XMo0	95.99	4.01	0.00
XMo1	90.35	8.06	1.59
XMo6	93.03	4.56	2.41
XMo14	85.29	8.46	6.24

**Table 3:** Kinetic current density, number of electrons transferred and peroxide selectivity for the prepared materials.

Sample	*n*_*KL*, (−0.8V)_	$J_{\begin{array}{c} KL\left(-0.8V\right)\\ \mathrm{mA}/{cm}^{2} \end{array}}$	$n_{\left(-0.8V\right)}$ RRDE	$\%H_{2}O_{2(-0.8V)}$
XMo0	2.69	4.58	2.78	61.09
XMo1	3.57	11.03	3.35	32.38
XMo6	3.61	9.90	2.92	53.92
XMo14	2.32	6.20	2.67	66.48

## Data Availability

The raw data supporting the conclusions of this article will be made available by the authors on request.

## Supplementary Materials

The following supporting information can be downloaded at: https://www.mdpi.com/article/10.3390/gels12070617/s1. Figure S1: XPS spectra for the XMo0 sample; Figure S2: XPS spectra for the XMo1 sample; Figure S3: XPS spectra for the XMo14 sample; Figure S4: K-L dependence on plots for all samples; Figure S5: Adsorption isotherms for the prepared xerogels; Table S1: Binding energies obtained for All prepared samples; Table S2: Studies in literature reporting electrochemical characterization.

## Abbreviations

The following abbreviations are used in this manuscript:

TTC	Tetracycline Hydrochloride
ORR	Oxygen Reduction Reaction
AOPs	Advanced Oxidation Processes
SEM	Scanning Electron Microscopy
XMoY	Molybdenum–Carbon Xerogel Composite Containing Y Metal Weight Percentage
HRTEM	High-Resolution Transmission Electron Microscopy
HAADF	High-Angle Annular Dark Field
TGA	Thermogravimetric Analysis
