# Supplementary material for: Molybdenum–Carbon Xerogel Composites for ORR-Based Electro-Catalytic Applications
Source: Gels. 2026 Jul 9;12(7):617. doi: 10.3390/gels12070617 (PMC13409661; doi:10.3390/gels12070617)
Supplement: Supplementary file 1 [file gels-12-00617-s001.zip › gels-4352234-supplementary.pdf]

Supplementary Data

Table S1. Binding energies obtained for All prepared samples

Sample	C1s			O1s			Mo 3d		
XMo0	B.E., eV	Bonds	%Área	B.E., eV	Bonds	%Área	B.E., eV	Bonds	%Área
	284.6	C = C sp^2	57.29	531.6	C = O	28			
	285.6	C - C sp^3	23.38	533.0	O - C, C - OH, C - O - C	57			
	286.9	R - OH	7.68	534.7	Chemisorbed H_2O	15			
	288.6	C = O	5.09						
	290.2	COOH	4.58						
	291.6	π plasmon	1.98						
XMo1	284.6	C = C sp^2	61.52	531.6	C = O	45			
	285.2	C - C sp^3	20.80	533.0	O - C, C - OH, C - O - C	40			
	286.1	R - OH	7.49	534.7	Chemisorbed H_2O	15	232.8	MoO_3 $Mo3d_{5/2}$	62
	288.1	C = O	4.29				236.0	MoO_3 $Mo3d_{3/2}$	38
	289.9	COOH	4.20						
	291.3	π plasmon	1.40						
XMo6	284.6	C = C sp^2	58.80	531.6	C = O	43	228.7	Mo_2C $Mo3d_{5/2}$	4
	285.2	C - C sp3	22.05	533.0	O - C, C - OH, C - O - C	40	231.8	Mo_2C $Mo3d_{3/2}$	3
	286.5	R - OH	7.45	534.7	Chemisorbed H_2O	17	232.7	MoO_3 $Mo3d_{5/2}$	59
	288.1	C = O	5.03				235.9	MoO_3 $Mo3d_{3/2}$	34
	289.9	COOH	4.30						
	291.5	π plasmon	1.97						
XMo14	284.6	C = C sp^2	58.69	531.6	C = O	56			
	285.6	C - C sp^3	20.41	533.0	O - C, C - OH, C - O - C	28			
	286.9	R - OH	7.94	534.7	Chemisorbed H_2O	16	232.8	MoO_3 $Mo3d_{5/2}$	63
	288.6	C = O	6.11				236.0	MoO_3 $Mo3d_{3/2}$	37
	290.2	COOH	2.99						
	291.6	π plasmon	1.86						

Table S2. Studies in literature reporting electrochemical characterization.

Sample	Kinetic density (mA/cm^2)	Electrons transferred	%H_2O_2	Ref
Mesoporous carbon (MC)	1.68	2.26	NR	[1]
Mo-MC	2.63	2.31		
MC-Graphene composite (MCG)	4.27	2.83		
Mo-MCG	4.95	3.56		
Mo_2C-supported Carbon Xerogel	2.9	3	NR	[2]
Mo_2C-supported Carbon nanotubes	1.6	2.7		
Hollow Mo microspheres	4.2	3.2 - 3.6	NR	[3]
(Fe,Mo) - N/C	NR	4	NR	[4]
Mo_2C supported-N-doped nanofibers	2.3	2.1 - 3.2	NR	[5]
XMo1	11.03	3.35	32.38	This Work
XMo6	9.9	2.92	53.92	
XMo14	6.2	2.67	66.48	

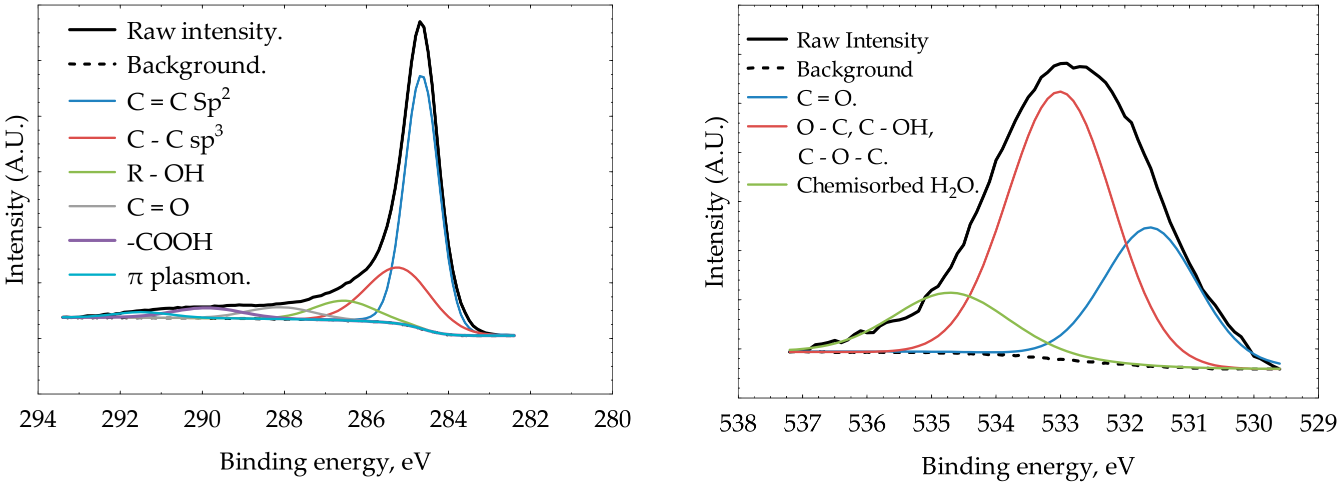

XMo0: C1s

XMo0: O1s

Figure S1. XPS spectra for the XMo0 sample.

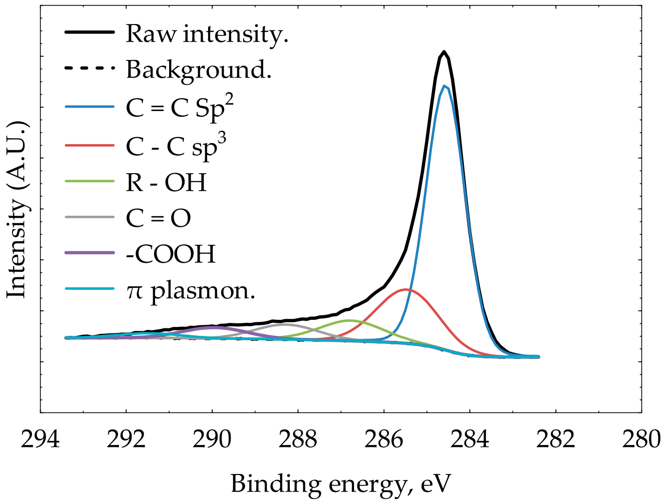

XMo1: C1s

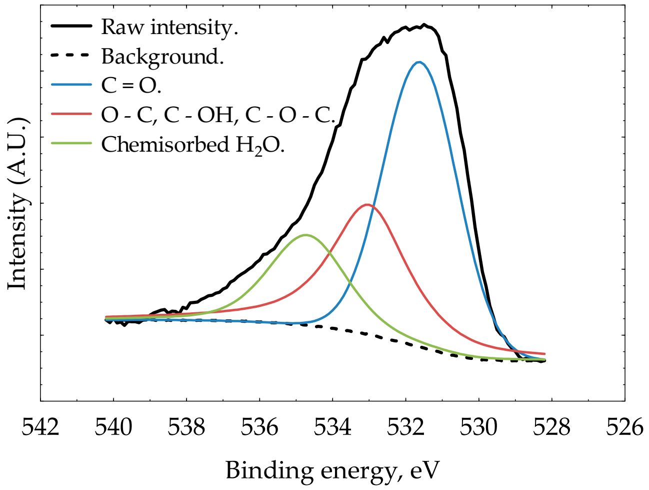

XMo1: O1s

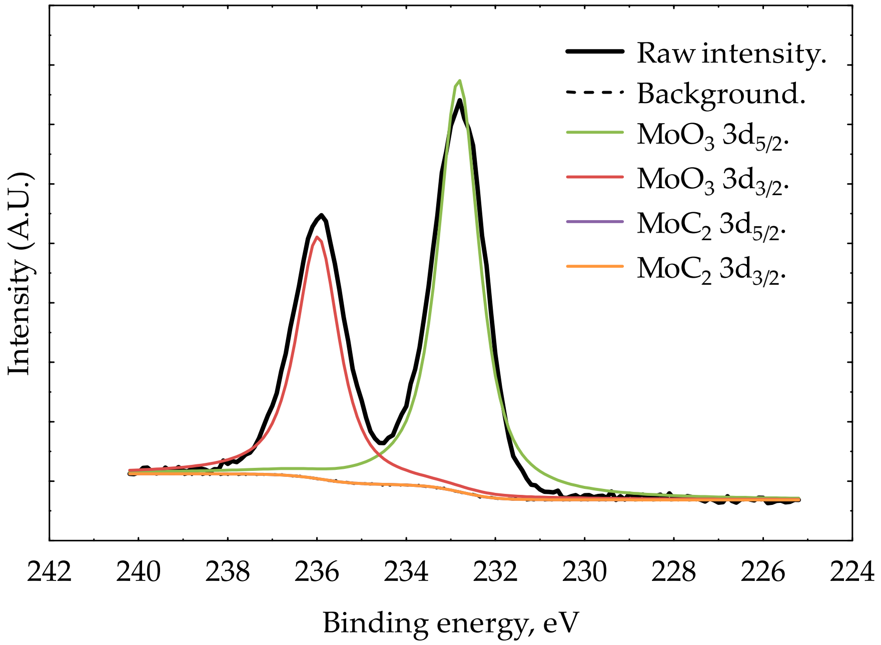

XMo1: Mo3d

Figure S2. XPS spectra for the XMo1 sample.

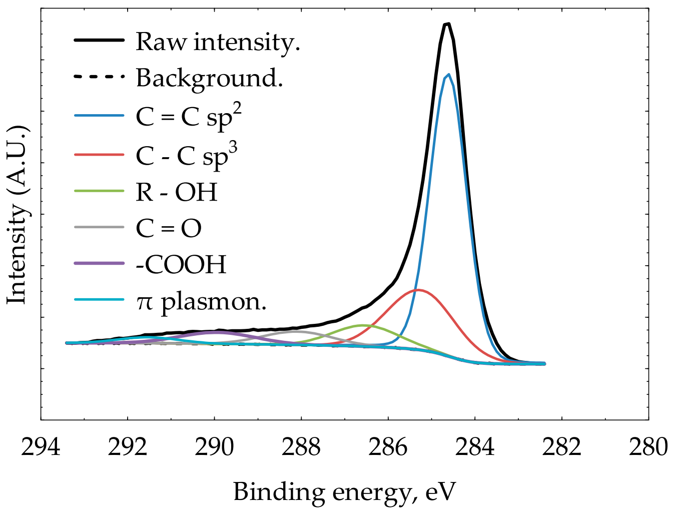

XMo14: C1s

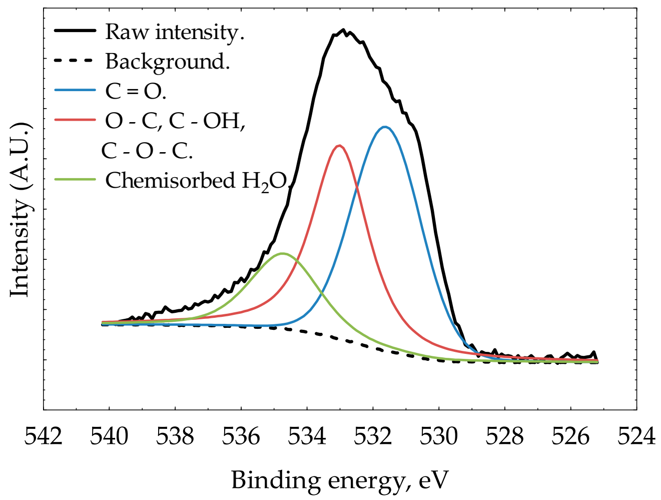

XMo14: O1s

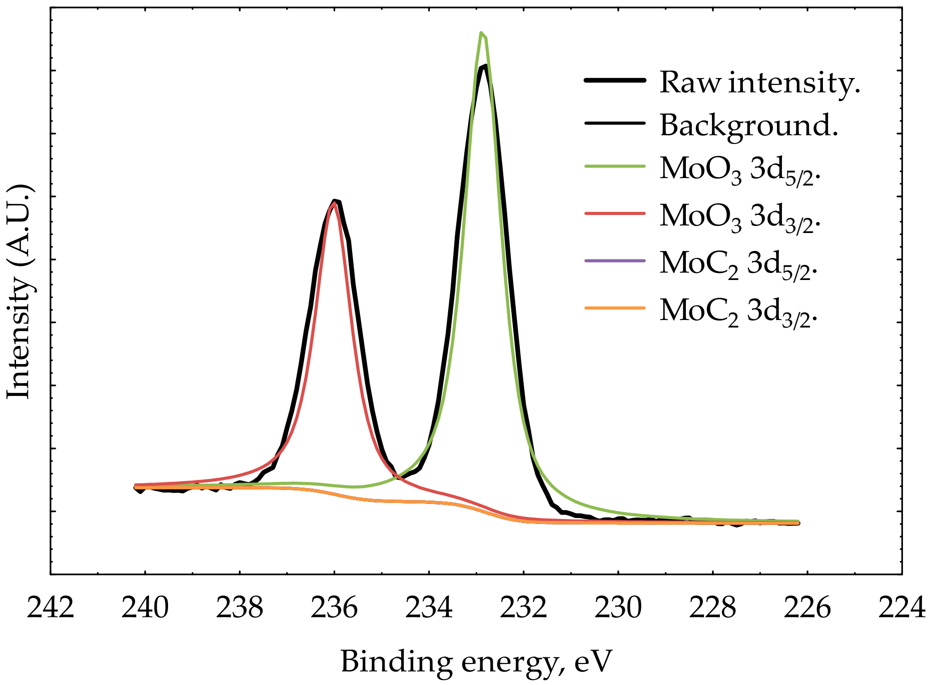

XMo14: Mo3d

Figure S3. XPS spectra for the XMo14 sample.

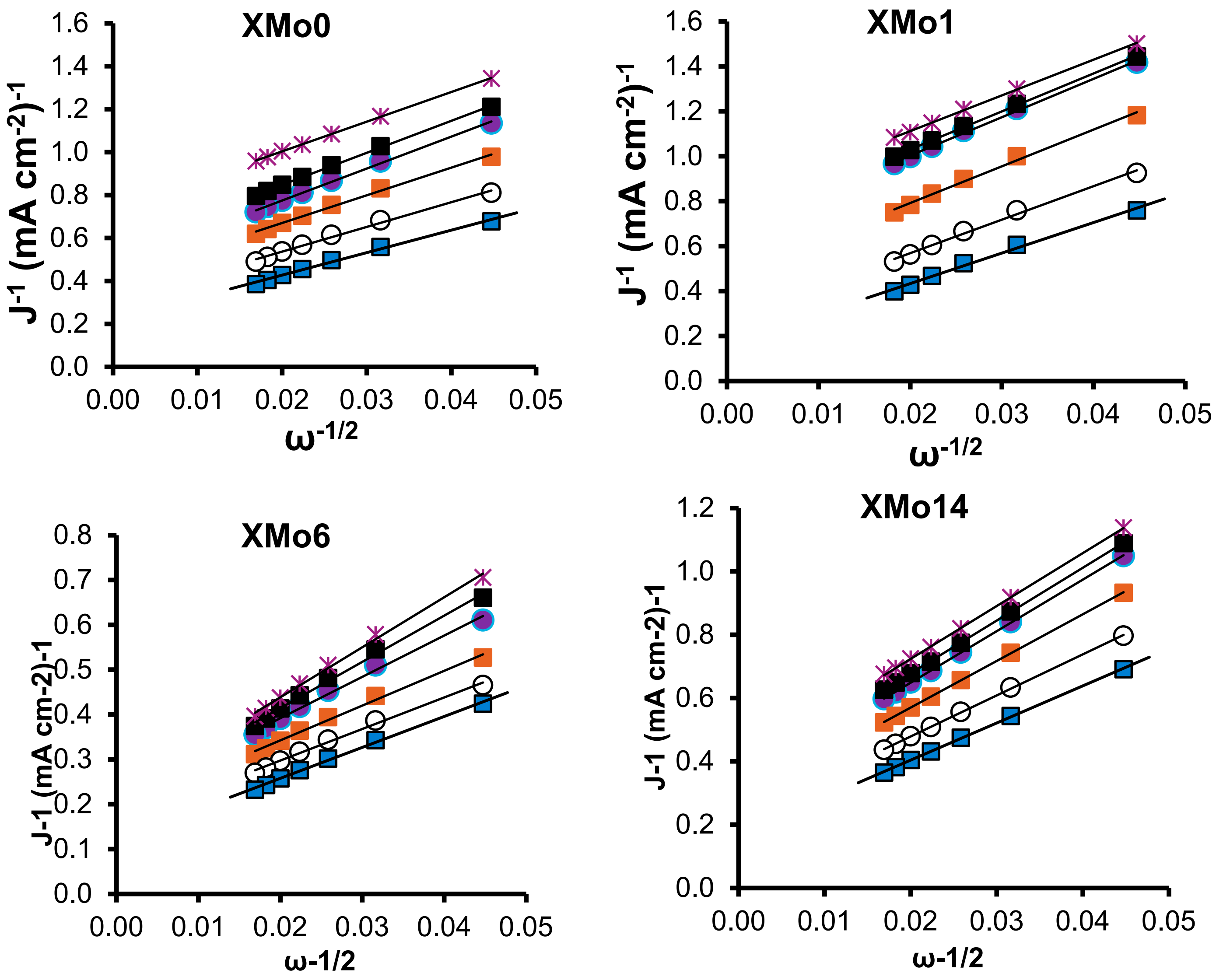

Figure S4. K-L dependence on plots for all samples.

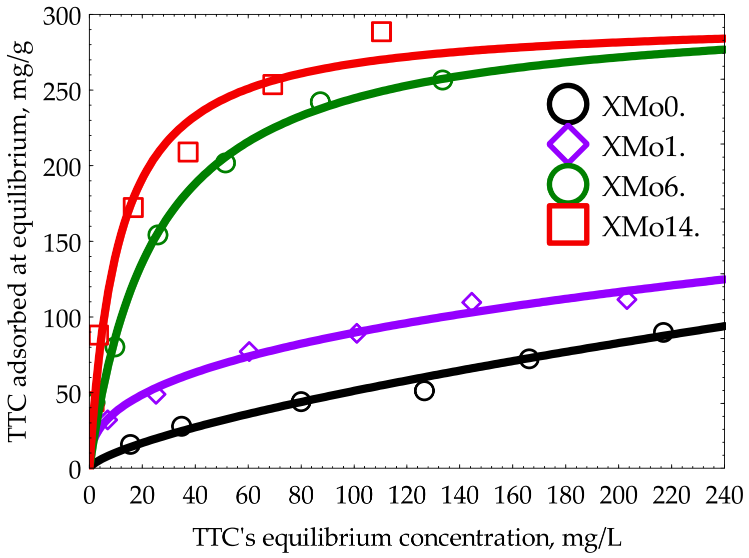

Figure S5. Adsorption isotherms for the prepared xerogels. C_0 = 25 – 250 mg/L. V/m = 2, pH = 6.5, T = 25 °C. Lines correspond to a fit to the Langmuir isotherm.

1. Dong, Y.; Liu, M.; Liu, Y.; Wang, S.; Li, J. Molybdenum-Doped Mesoporous Carbon/Graphene Composites as Efficient Electrocatalysts for the Oxygen Reduction Reaction. *J. Mater. Chem. A Mater.* **2015**, *3*, 19969–19973, doi:10.1039/c5ta04624f.
2. Mladenovic, D.; Vujkovic, M.; Mentus, S.; Santos, D.M.F.; Rocha, R.P.; Sequeira, C.A.; Figuereido, J.-L.; Sljukic, B. Carbon-Supported Mo2C for Oxygen Reduction Reaction Electrocatalysis. *Nanomaterials* **2020**, 1–12, doi:10.3390/nano10091805.
3. Luo, Y.; Wang, Z.; Fu, Y.; Jin, C.; Wei, Q.; Yang, R. In Situ Preparation of Hollow Mo2C–C Hybrid Microspheres as Bifunctional Electrocatalysts for Oxygen Reduction and Evolution Reactions. *J. Mater. Chem. A* **2016**, *4*, 12583–12590, doi:10.1039/C6TA04654A.
4. Lin, L.; Yang, Z.K.; Jiang, Y.; Xu, A. Nonprecious Bimetallic (Fe,Mo) − N/C Catalyst for E Ffi Cient Oxygen Reduction Reaction. *ACS Catal.* ***2016**, 6, 7, 4449–4454 doi:10.1021/acscatal.6b00535.*
5. Wang, H.; Sun, C.; Cao, Y.; Zhu, J.; Chen, Y.; Guo, J.; Zhao, J.; Sun, Y.; Zou, G. Molybdenum Carbide Nanoparticles Embedded in Nitrogen-Doped Porous Carbon Nano Fi Bers as a Dual Catalyst for Hydrogen Evolution and Oxygen Reduction Reactions. *Carbon N. Y.* **2017**, *114*, 628–634, doi:10.1016/j.carbon.2016.12.081.
